# Regional variations in the treatment of gallstone disease may affect patient outcome: a large, population-based register study in sweden

**DOI:** 10.1177/1457496920968015

**Published:** 2020-10-27

**Authors:** Lisa Lindqvist, Gabriel Sandblom, Pär Nordin, Oskar Hemmingsson, Lars Enochsson

**Affiliations:** Department of Surgical and Perioperative Sciences, Surgery, Umeå University, Umeå, Sweden; Surgery, Department of Clinical Science and Education, Södersjukhuset, Karolinska Institutet, Stockholm, Sweden; Department of Surgical and Perioperative Sciences, Surgery, Umeå University, Umeå, Sweden; Department of Surgical and Perioperative Sciences, Surgery, Umeå University, Umeå, Sweden; Professor of Surgery, Department of Surgical and Perioperative Sciences, Surgery, Umeå University, Umeå, SE-901 87, Sweden

**Keywords:** Gallstone, cholecystectomy, cholecystitis, regional differences, population density, complications

## Abstract

**Background:**

The lack of studies showing benefit from surgery in patients with symptoms of gallstone disease has led to a divergence in local practices and standards of care. This study aimed to explore regional differences in management and complications in Sweden. Furthermore, to study whether population density had an impact on management.

**Methods:**

Data were collected from the Swedish National Register for Gallstone Surgery and Endoscopic Retrograde Cholangiopancreatography (GallRiks). Cholecystectomies undertaken for gallstone disease between January 2006 and December 2017 were included. Age, sex, American Society of Anesthesiologists (ASA) classification, intra- and post-operative complications, and the proportion of patients with acute cholecystitis who underwent surgery within 2 days of hospital admission were analyzed. The 21 different geographical regions in Sweden were compared, and each variable was analyzed according to population density.

**Results:**

A total of 139,444 cholecystectomies cases were included in this study. There were large differences between regions regarding indications for surgery and intra- and post-operative complications. In the analyses, there were greater divergences than would be expected by chance for most of the variables analyzed. Age of the cholecystectomized patients correlated with population density of the regions (R^2^ = 0.310; p = 0.0088).

**Conclusions:**

There are major differences between the different regions in Sweden in terms of the treatment of gallstone disease and outcome, but these did not correlate to population density, suggesting that local routines are more likely to have an impact on treatment strategies rather than demographic factors. These differences need further investigation to reveal the underlying causes.

## Introduction

Gallstone disease is common in the Western world. In a Swedish population study including 739 subjects (35–85 years) screened for gallstone/cholesterolosis/sludge with ultrasonography, the overall prevalence of a positive finding was 16.3%^1^. The estimated incidence of gallstone disease in the Swedish population is 1.39 per 100 person-years^2^.

The majority of patients with symptomatic gallstone disease experience biliary colic but a large portion also develops complicated gallstone disease, that is, acute cholecystitis, biliary pancreatitis, or obstructive jaundice^3^. Symptomatic gallstone disease is treated by cholecystectomy, that is, removal of the gallbladder, using one of the three techniques: laparoscopic, small-incision, or open cholecystectomy, of which laparoscopic cholecystectomy has become the gold standard^4^. Cholecystectomy is one of the most common general surgical operations, with approximately 13,000 operations performed annually in Sweden^5^.

Acute cholecystitis is a common secondary complication to gallstone disease and affects 20% of patients admitted to the hospital for biliary tract disease^6,7^. In Sweden, 60% of patients with acute cholecystitis are treated surgically in the acute phase of the disease. If the proportion operated on in the acute stages increased to 90%, three hospital care days per patient would be saved^8^. The current recommendation is surgery within 5 days or after 3 months. However, surgery within 2 days of admission for acute cholecystitis considerably reduces the risk for intra- and post-operative complications as well as reducing mortality^9,10^.

While the benefit of early surgical intervention is well established for acute cholecystitis, research on patients with mild to moderate symptoms is limited. This has led to divergent standards of care, where the decision to perform surgery largely depends on local practice as well as the surgeon responsible. How this affects the time and rate of surgery has been described previously^11^, but there are few studies on whether regional administrative and geographical circumstances affect operative incidence. Surgical treatment in a timely fashion is crucial for reducing morbidity and the risk for adverse outcomes associated with acute cholecystitis.

It has been shown that patients who live far away from major hospitals with their associated cancer centers have a lower chance of survival from lung or colorectal cancer, as their disease is more advanced upon diagnosis^12^. We thus hypothesized that patients who live in less populated rural areas and are required to travel long distances to the nearest major hospital, as is often the case in the north of Sweden, wait longer before they seek medical attention for an illness. Furthermore, patients in rural areas are treated differently than their urban counterparts in more densely populated parts of Sweden because of the demographics and long traveling distances to the nearest healthcare facility.

**Fig. 1.**
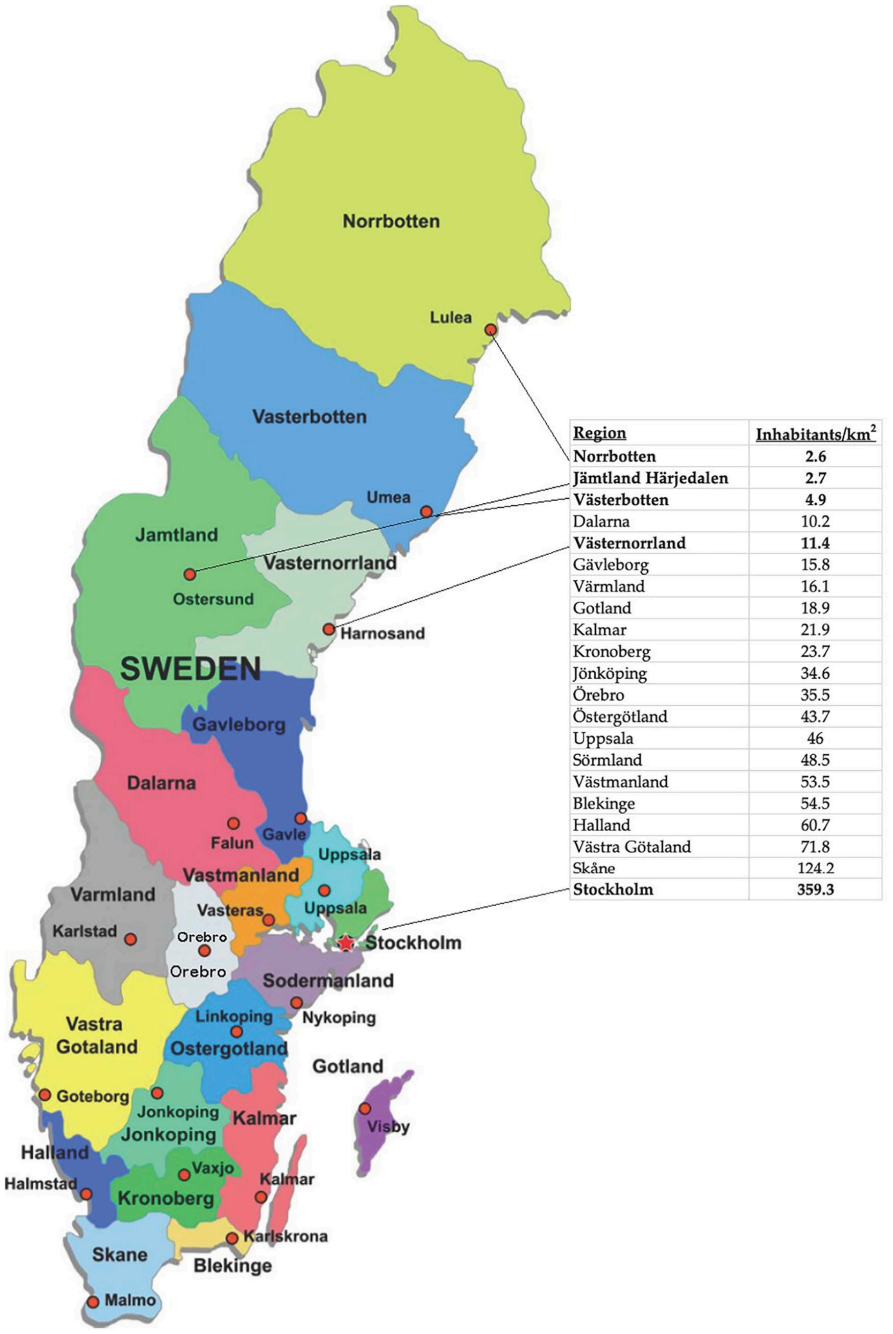
The 21 regions of Sweden and their population densities.

Sweden is divided into 21 geographical and administrative regions that vary greatly in population density; from 2.6 to 359.3 inhabitants per square kilometer (Fig. 1) with an average of 25.1 inhabitants per square kilometer, a figure much lower than the European Union average of 117.7^13,14^. Age distribution is another factor that varies greatly between regions as a result of rural depopulation. Mean age in the three regions with the highest population densities (39.4, 41.1, and 40.9 years, respectively) is lower than in the region with the lowest population density (44.0 years)^15^. Thus, provision of equal healthcare despite demographic differences between regions represents a great challenge. The aim of this study was to investigate regional inequity in the management of gallstone disease and whether region-based differences correlate to population density.

## Materials and Methods

This is a population-based register study. Data were collected from the Swedish National Quality Register for Gallstone Surgery and Endoscopic Retrograde Cholangiopancreatography (GallRiks). The register was founded in 1 May 2005 and currently covers 90% of all cholecystectomies performed in Sweden^16^. Each year, around 13,000 cholecystectomies are added to the register^5,17^. Cholecystectomy registration is performed online by the surgeon directly after the operation via secure smartcard login on the GallRiks website. A 30-day follow-up is mandatory and performed by the local coordinator at each participating unit. He or she looks through the medical records of each operated patient, and if necessary, also contacts the patients by phone so as not to miss any post-operative complication.

The data on cholecystectomies performed between 1 January 2006 and 31 December 2017 were obtained from the GallRiks register. Operations performed for reasons other than gallstone disease (e.g. suspected malignancy or as part of major surgery) were excluded. The main indication for surgery was stated by the surgeon in all cases of the cholecystectomy performed for gallstone disease.

Cholecystectomies performed in the 21 Swedish geographical and administrative regions were compared. The variables analyzed were the main indication for surgery (i.e. colic or complicated gallstone disease) and intra- and post-operative complications. Patient-related factors including age, sex, and American Society of Anesthesiologists (ASA) classification were also included. These variables were also analyzed according to the population density of each region.

### Statistical Analyses

All statistical analyses were performed using JMP Pro (JMP^®^, Version 14.2.0; SAS Institute Inc., Cary, NC, USA). The funnel plots show the proportion of data from each region and the 95% and 99.8% confidence interval (CI) limits.

### Ethical Considerations

All data were anonymized, and all analyses were conducted at the group level. This study was approved by the Ethics Committee of Umeå University, Umeå, Sweden (Dnr 2019-00138) and was conducted in accordance with the ethical principles outlined in the Declaration of Helsinki.

## Results

**Fig. 2.**
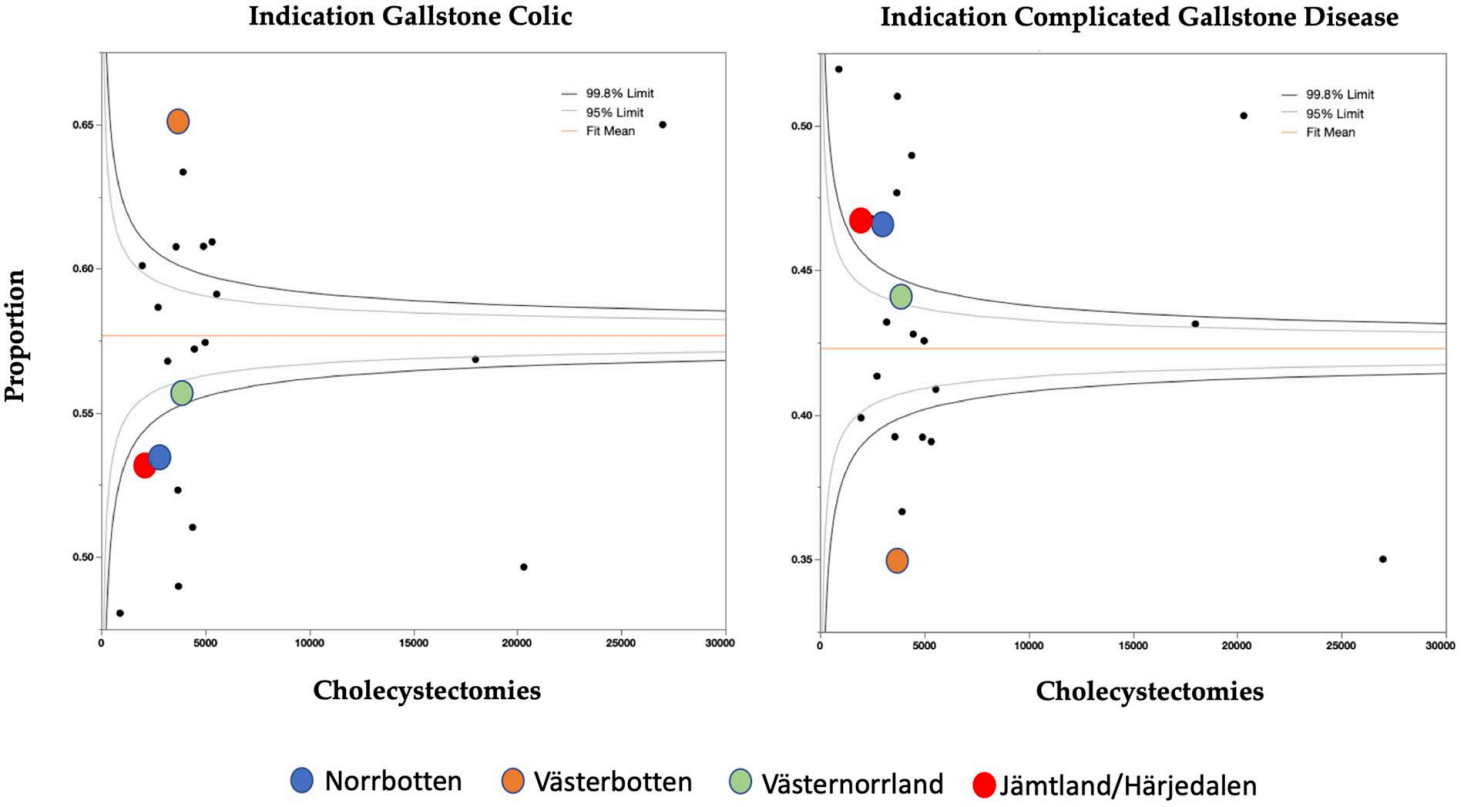
Funnel plot of “gallstone colic” and “complicated gallstone disease” as indication per region. The four most northern, least populated regions in Sweden are marked with circles of different colors.

Between January 2006 and 31 December 2017, a total of 139,444 cholecystectomies were registered in GallRiks. Of these, 8041 were excluded due to indications other than biliary colic or complicated gallstone disease (cholecystectomy part of major surgery 1.9%, suspected malignancy/polyp 1.4%, indication missing 1.1%, unspecified indication 0.7%, acalculous cholecystitis 0.5%), leaving 131,403 cholecystectomies of which 75,807 (58%) underwent surgery for “gallstone colic” and the remaining 55,596 (42%) for “complicated gallstone disease.” Patient demographic data—including sex, ASA classification, and age (50.6 ± 16.3 years; mean (standard deviation (SD))), dichotomized into ⩽50 years and >50 years, respectively—are presented according to region as well as indication in Table 1. Of the cases with the indication “gallstone colic,” females predominated (75% vs 25%) whereas in those with the indication “complicated gallstone disease,” the gender difference was less pronounced (55 vs 45%). In the latter category, there were also greater variations in gender distribution between the different regions, whereas in those with the indication “gallstone colic,” gender distribution was similar throughout the different regions (Table 1). The patients in the “complicated gallstone disease” group had higher ASA scores and were older (55.0 ± 16.7 vs 47.3 ± 15.2 years; mean (SD), p < 0.0001) compared to the group undergoing surgery for “gallstone colic.” The group with “complicated gallstone disease” was more heterogeneously distributed between the regions (Table 1). As seen in the two Funnel plots in Fig. 2, the indications of gallstone colic and complicated gallstone disease show a heterogeneous pattern between the regions. The region Västerbotten, where the tertiary center of northern Sweden is located, shows a somewhat different pattern compared to the other three northern regions. These regions are marked with circular markers in different colors (Fig. 2).

**Table 1.** Sex, ASA classification, and age (dichotomized into ⩽50 years and >50 years, respectively) for each respective region and divided into the indications gallstone colic and complicated gallstone disease.

Regions	Gallstone colic							Complicated gallstone disease						
	n	Females		ASA I and II		⩽50 years^a^		n	Females		ASA I and II		⩽50 years^b^	
		n	%	n	%	n	%		n	%	n	%	n	%
Blekinge	1181	887	75	1151	97	630	53	784	427	55	692	88	241	31
Dalarna	2242	1702	76	2151	96	1258	56	2151	1186	55	1911	89	751	35
Gotland	432	324	75	412	95	222	51	467	272	58	407	87	154	33
Gävleborg	2492	1837	74	2386	96	1333	54	1442	775	54	1285	89	524	36
Halland	1818	1418	78	1719	95	1046	58	1893	1064	56	1683	89	675	36
**Jämtland Härjedalen**	**1308**	**962**	**74**	**1253**	**96**	**737**	**56**	**1150**	**591**	**51**	**968**	**84**	**350**	**30**
Jönköping	3279	2490	76	3136	96	1931	60	2268	1211	53	1948	86	799	35
Kalmar	1927	1469	76	1855	96	1096	57	1756	974	55	1562	89	592	34
Kronoberg	1602	1206	75	1561	97	914	57	1129	605	54	1010	89	429	38
**Norrbotten**	**1530**	**1160**	**76**	**1430**	**93**	**807**	**53**	**1337**	**711**	**53**	**1109**	**83**	**487**	**37**
Skåne	10,231	7842	77	9738	95	5924	58	7763	4369	56	6842	88	2938	38
Stockholm	17,540	12,956	74	16,143	92	10,394	60	9449	5225	55	8186	87	4269	46
Sörmland	1814	1355	75	1740	96	1045	58	1380	748	54	1246	90	511	37
Uppsala	2868	2086	73	2684	94	1706	60	2125	1139	54	1782	84	904	43
Värmland	2183	1673	77	2017	92	1225	56	1410	768	55	1196	85	483	34
**Västerbotten**	**2398**	**1760**	**73**	**2227**	**93**	**1317**	**55**	**1287**	**649**	**50**	**1096**	**85**	**484**	**38**
**Västernorrland**	**2082**	**1521**	**73**	**2022**	**97**	**1206**	**58**	**1654**	**869**	**53**	**1469**	**89**	**659**	**40**
Västmanland	2559	1951	76	2392	93	1535	60	1914	1088	57	1664	87	778	41
Västra Götaland	10,086	7739	77	9678	96	5968	59	10,226	5638	55	9223	90	4044	40
Örebro	2987	2235	75	2894	97	1698	57	1928	1055	55	1785	93	760	40
Östergötland	3248	2460	76	3150	97	1961	60	2083	1177	57	1836	88	746	36
Total	**75,807**	57,033	75	71,739	95	43,953	58	**55,596**	30,541	55	48,900	88	21,578	39

ASA: American Society of Anesthesiologists.

The four most Nordic regions in Sweden are marked in bold.

a 133 where age is not stated.

b 167 where age is not stated.

Age correlated significantly with population density (R^2^ = 0.310; p = 0.0088). In regions with higher population densities, the patients tended to be younger at the time of surgery.

**Fig. 3.**
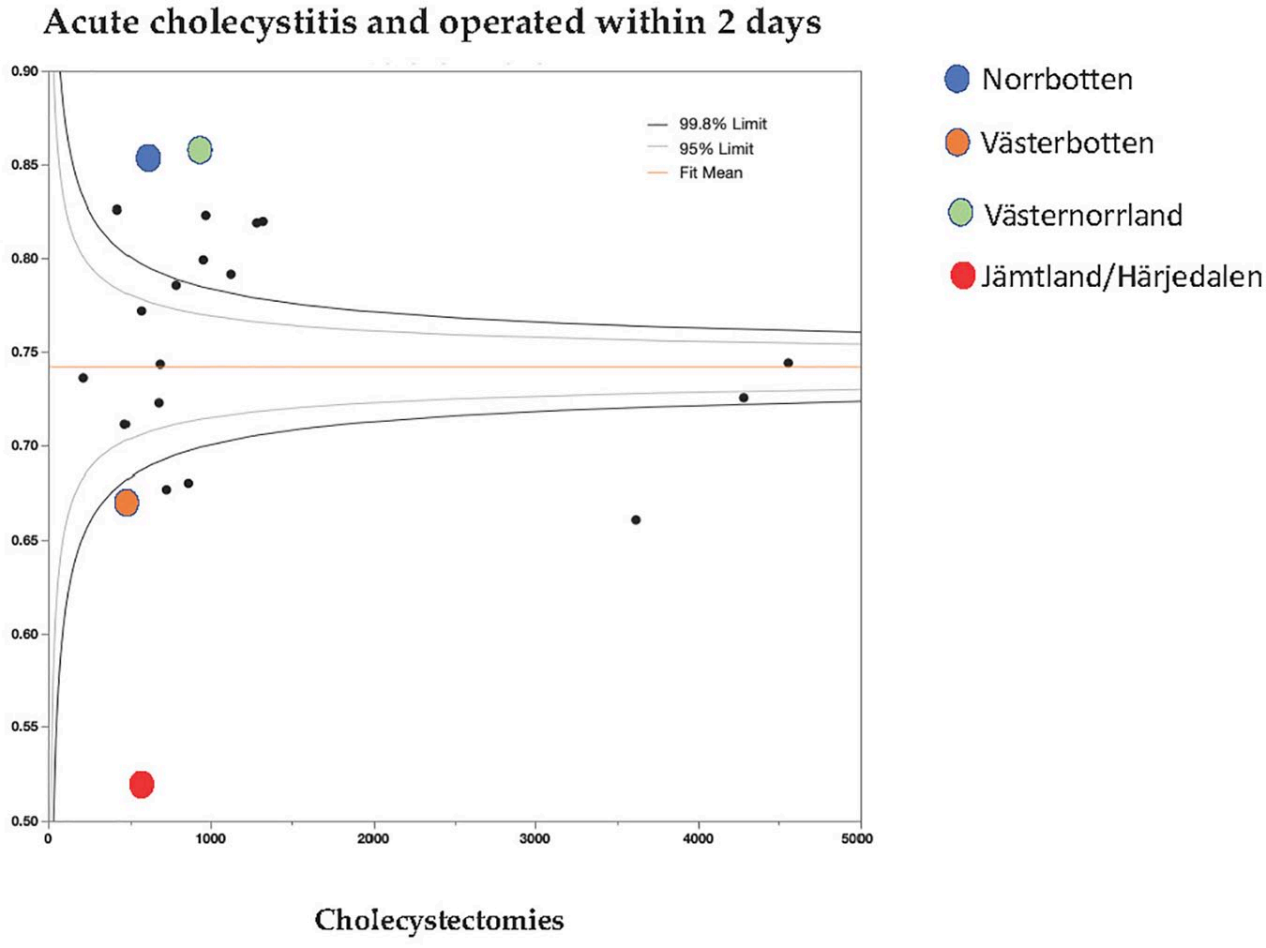
Funnel plot of patients with acute cholecystitis operated within 2 days of admission according to region. The four most northern least populated regions are marked with circles of different colors.

Of the 55,596 with the indication “complicated gallstone disease,” 26,107 (47%) were judged by the operating surgeon to have ongoing acute cholecystitis, 8.1% pancreatitis, and 20.8% increased bilirubin and/or common bile duct stones (CBDSs). Fig. 3 shows the differences between regions regarding the proportion of patients with ongoing acute cholecystitis operated within 2 days of admission. A high degree of variability was revealed by this subgroup analysis. Eight regions deviated above the 99.8% funnel plot limit and five below the 99.8% CI limit. Six of the 21 regions were within the 95% CI limit. Although two of the four least populated northern Swedish regions were significantly below the 99.8% CI limit, there was no correlation between population density and acute cholecystitis requiring surgery within 2 days of admission (p = 0.6439). In contrast, the rate of surgery for cholecystitis within 2 days after admission in the other two least populated regions was much higher (Fig. 3).

**Fig. 4.**
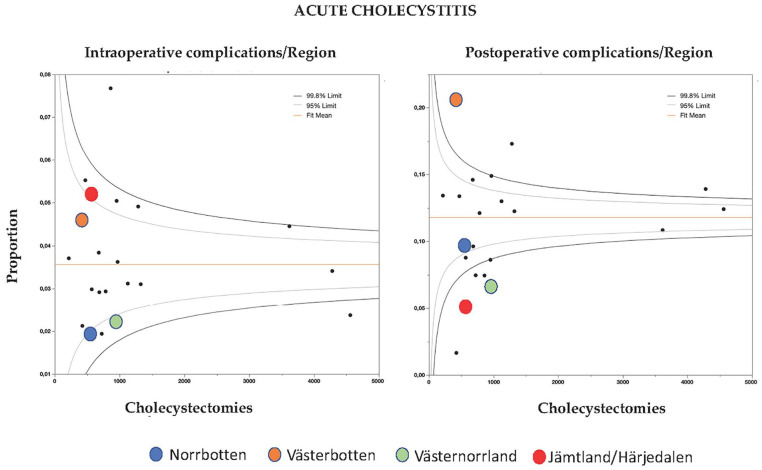
Funnel plot of intra-operative (left) and post-operative complications (right) per region after cholecystectomy for acute cholecystitis. The four most northern least populated regions are marked with circles of different colors.

The incidence of intra-operative complications in patients with acute cholecystitis varied from 1.9% to 7.7% between the different regions. However, the intra-operative complication rates were relatively well aggregated with only two regions outside the 99.8% limit (Fig. 4). Post-operative complication rates, however, were more heterogeneously distributed with eight regions outside the 99.8% CI limit, ranging from 1.6% to 20.7% (Fig. 4). The four northern regions also varied regarding post-operative complications, where two had a distribution below and one above the 99.8% limit.

**Fig. 5.**
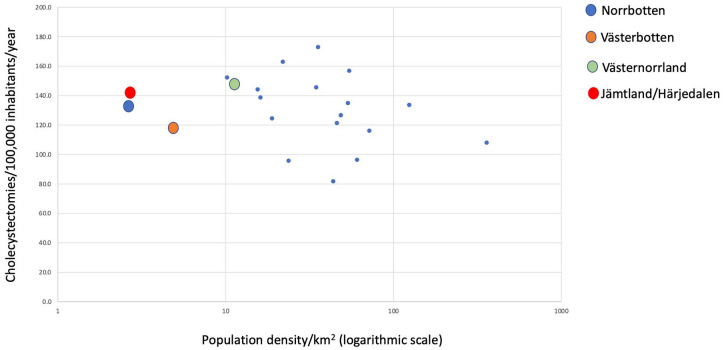
Logarithmic plot of population density/km^2^ (x-axis) versus cholecystectomies/100,000 inhabitants/year.

However, neither intra- nor post-operative complications correlated to population density. There was no statistically significant correlation between population density and the annual cholecystectomy rate per 100,000 inhabitants, which ranged from 81.7 to 173.0 (R^2^ = 0.08; p = 0.2193) (Fig. 5). The four most northern regions in Sweden ranged from 117 (Västerbotten) to 148 (Västernorrland) cholecystectomies/100,000 inhabitants/year.

## Discussion

This is a register study on a large cohort of 131,403 cholecystectomies performed in Sweden. Comparison of the 21 regions in Sweden showed significant differences in terms of both patient demographics and treatment policies. Our original hypothesis that population density correlates with indication for and outcome of cholecystectomy was not supported by the data. The only study variable that correlated with population density was age at time of cholecystectomy, where patients in low density regions tend to be older at the time of surgery. This may be explained by well-known age distribution patterns in urban and rural populations.

The beneficial effects of early intervention in patients with acute cholecystitis have been shown in several studies^9,10^. We did not find any correlation between population density and the proportion undergoing a cholecystectomy within 2 days in patients with acute cholecystitis. However, two of the least populated regions in northern Sweden had the lowest rates of cholecystectomy on patients with acute cholecystitis within 2 days of admission (Fig. 3). Our study indicates that local treatment policies may play a more important role than population density. In the group of patients operated for acute cholecystitis within 2 days of admission, the differences were quite pronounced, but this did not correlate significantly in a linear fashion to population density.

The limited evidence regarding surgery for gallstone disease in patients with mild to moderate symptoms has resulted in the absence of uniform consensus on optimal clinical management. This has led to differences in standards of care, where local routines seem to determine the treatment course, rather than evidence-based practice. Local routines depend to some extent on unavoidable factors such as infrastructure, resources, hospital size, and access to theater, but also the preferences of the surgeon. A good example of how local routines affect healthcare is the vast differences seen between regions in performing cholecystectomies on patients with acute cholecystitis within 2 days of admission.

It is well-known that healthcare resources are unevenly distributed between urban and rural areas and that it may be difficult to provide the same quality of care in sparsely populated areas as in large cities. Variation in ability to provide surgery to patients with gallstone disease was not reflected in regional differences in this study. Nevertheless, the study did show that there is a need for standardization of the management of patients with gallstone disease in Sweden.

A major strength of this study is the vast amount of validated data obtained from the GallRiks database^17,18^. Another strength is that the surgeon enters the patient’s data into GallRiks’ online register prospectively and surgical data immediately after cholecystectomy. Furthermore, GallRiks database has a high degree of coverage in that 90% of all cholecystectomies performed in Sweden are registered^16^.

There are some limitations to this study. One is the risk of bias regarding the registration of intra- and post-operative complications. This risk, to some extent, is negated by the 30-day follow-up by the independent local coordinator at each participating unit in GallRiks. Even so, the risk of bias regarding the registration of post-operative complications remains, since clinics that are the most diligent are at risk of having the highest complication rates^19^. The intra-operative complication rates of cholecystectomy for acute cholecystitis were low in all of Sweden’s 21 regions. However, post-operative complication rates varied, ranging from 1.6% to 20.7% with an average of 11.8% during the period of the study. The large spread in post-operative complication rates may be the result of uncertainty when recording complications.

A further limitation of this study is that we analyzed the different regions from a population density perspective only. There are many other perspectives that warrant analysis such as the fact that the four most northern regions of Sweden are served by only one tertiary center in the region of Västerbotten. A different picture was seen in Västerbotten regarding indications (Fig. 2) as well as post-operative complications (Fig. 4) when compared to the other three northern regions. These findings suggest that regional organization and hospital distribution within the four regions have a greater impact on the indications for and outcome of cholecystectomy for acute gallstone disease than population density.

We adjusted for some potential confounders. There are, however, other factors such as smoking, body mass index (BMI), and alcohol consumption that may vary between the regions. These factors influence not only the development of cholecystolithiasis but also the outcome after surgery for gallstone disease. We cannot rule out that these confounders are unevenly distributed between regions in Sweden, a fact that could have affected the outcome of this study. Unfortunately, during the study period, only BMI was registered in GallRiks and since this was introduced as late as 2010, we did not include BMI in the present analyses. Neither smoking nor drinking was registered during the time of the study.

In conclusion, there are large regional differences in Sweden regarding demographic factors, indications for gallstone surgery, and complication rates after cholecystectomy. Only age correlated significantly in a linear fashion to population density despite the variation of 2.6–359.3 inhabitants per square kilometer between regions. The findings in this study indicate that local routines rather than established international guidelines^20^ have greatest impact on the treatment of gallstone disease in Sweden. Furthermore, apart from age, demographic factors, including population density, seem to have little impact on the management of this disease at the group level. Further large register-based studies employing Geographic Information Systems ^21^, as a more precise method for analyzing demographic factors, may establish whether access to healthcare facilities affects patient outcome at the individual level.
